# Long-term results of treatment with bosentan in adult Eisenmenger’s syndrome patients with Down’s syndrome related to congenital heart disease

**DOI:** 10.1186/1471-2261-13-74

**Published:** 2013-09-18

**Authors:** Roberto Crepaz, Cristina Romeo, Donato Montanaro, Stefano De Santis

**Affiliations:** 1 Department of Cardiology and Pediatric Cardiology, Regional Hospital of Bolzano, Bolzano, Italy; 2 Medical Department, Actelion Pharmaceuticals Italia Srl, Imola, Italy; 3 Cardiologia e Prove funzionali/Kardiologie und kardiologische Funktionsproben, Ospedale Centrale di Bolzano/Krankenhaus Bozen, Via/Straße Lorenz Böhler 5, Bolzano/Bozen, 39100, Italy

**Keywords:** Bosentan, Eisenmenger’s syndrome, Down’s syndrome, Long-term

## Abstract

**Background:**

Patients with Down’s syndrome and shunt lesions are at high risk of developing pulmonary arterial hypertension (PAH) earlier than patients without Down’s syndrome. However, data on the efficacy of PAH-specific therapy in patients with Down’s syndrome are limited. The aim of this retrospective analysis was to determine the long-term efficacy of the dual endothelin receptor antagonist, bosentan, in Eisenmenger's syndrome (ES) patients with Down’s syndrome.

**Methods:**

In this observational study adults with Down’s syndrome with a confirmed diagnosis of ES (World Health Organization functional class III) and receiving bosentan therapy and were followed up long term. Clinical evaluation at baseline and follow-up visits included resting transcutaneous arterial oxygen saturation and laboratory assessments. Exercise capacity was evaluated using a 6-minute walk test where transcutaneous arterial oxygen saturation at peak exercise (SpO_2_), 6-minute walk distance (6MWD) and Borg dyspnoea index were assessed. A full echocardiographic assessment was conducted at baseline and follow-up visits.

**Results:**

Overall, seven adults (mean age 29.6 ± 11.2 years; 57% male) received bosentan at a starting dose of 62.5 mg twice daily. This was increased to the target dose of 125 mg twice daily 4 weeks later. All patients remained on bosentan until the end of the study. After a mean (± standard deviation) duration of 52.2 ± 3.9 months (range: 46.0–55.5 months), 6MWD had increased from 199.6 ± 69.1 metres to 303.7 ± 99.9 metres (*P* < 0.05) and SpO_2_ at the end of the 6-minute walk test had increased from 61.6 ± 7.6% to 74.7 ± 6.2% (*P* < 0.05). Echocardiography demonstrated a significant change in acceleration time from 62.9 ± 11.6 m/s to 83.0 ± 9.6 m/s (*P* = 0.0156), and acceleration time/ejection time ratio from the pulmonary flow from 0.24 ± 0.04 at baseline to 0.30 ± 0.02 (*P* = 0.0156) at final follow-up.

**Conclusions:**

Long-term treatment with bosentan significantly improved exercise capacity and oxygen saturation following exercise in adult ES patients with Down’s syndrome. These data confirm that the presence of Down’s syndrome does not affect the response to oral bosentan therapy.

## Background

Patients with congenital heart disease (CHD) with significant untreated left-to-right shunt lesions are at risk of developing pulmonary arterial hypertension (PAH). Eisenmenger’s syndrome (ES), a clinical condition with an initial large systemic-to-pulmonary shunt that induces severe pulmonary vascular disease [1], represents the most advanced form of PAH-CHD and is characterised by reversed pulmonary-to-systemic (right-to-left) shunt and central cyanosis. The Euro Heart Survey on adult CHD (a retrospective cohort study with a 5-year follow-up) reported PAH in 531 (28%) patients [2], while a Dutch registry showed a PAH prevalence of 4.2% in 5970 adult patients with CHD [3]. In the latter registry, 58% of patients with a septal defect who developed PAH had ES.

Patients with Down’s syndrome represent an important subgroup in the ES population as they are considered to be at risk of PAH earlier than patients without Down’s syndrome and also have worse functional capacity [4]. This is probably due to different pathogenetic factors between patients with and without Down’s syndrome [5]. Down’s syndrome has been estimated to occur in ≈ 1.1/1000 adults in the UK [6] and ≈ 1/732 infants in the USA [7]. However, there is some evidence that prevalence varies between different racial and ethnic groups [8]. The association between Down’s syndrome and CHD has been well established [9]. The prevalence of CHD in patients with Down’s syndrome is 40–60% [10,11] and the types of malformation that are most commonly associated with trisomy 21 are the atrio-ventricular and ventricular septal defects (AVSD and VSD), which together account for 76% of all CHD seen in patients with Down’s syndrome [12]. Although AVSD is frequently reported as the most common cardiac defect, there is some evidence that this varies according to sex and ethnicity [8,10-15].

Treatment options for ES patients are limited and the Bosentan Randomized Trial of Endothelin Antagonist Therapy-5 (BREATHE-5) study, which demonstrated the efficacy and tolerability of the endothelin receptor antagonist, bosentan, is the only placebo-controlled study in this population to date [16]. Data in patients with Down’s syndrome are even more limited, with the only evidence of the efficacy of PAH-specific therapies in this population being from small, or single-centre studies [17-22].

The aim of the present study was to evaluate the long-term efficacy of bosentan in adult ES patients with Down’s syndrome.

## Methods

This was a retrospective analysis of data collected from consecutive adult ES patients with Down’s syndrome (mild cognitive impairment verified by the Wechsler Adult Intelligence Scale – Revised [23]) treated with bosentan at our centre. Permission to collect data prospectively was obtained from the parents or the legal tutor prior to entering the study. Adults with a diagnosis of ES confirmed by right heart catheterization, in World Health Organization (WHO) functional class III between May 2007 and April 2008 were included. The final follow-up visits took place between January and February 2012.

Clinical evaluation at baseline included: medical history, WHO functional class, resting transcutaneous arterial oxygen saturation (SaO_2_) for 30 minutes and liver enzymes (alanine aminotransferase [ALT], aspartate aminotransferase [AST]). Exercise capacity was evaluated using a 6-minute walk test (6MWT) where the following variables were assessed: rest and peak exercise transcutaneous oxygen saturation (SpO_2_), heart rate, systemic blood pressure, 6-minute walk distance (6MWD) and Borg dyspnoea index. Several days prior to the 6MWT patients were educated about the 6MWT. The test was performed under the same environmental conditions and at approximately the same time of the day (± 1 hour) in all patients and physicians observed the patients’ attention and approach to the 6MWT.

A complete Doppler echocardiography (echo) study was conducted in each patient according to current European guidelines [24]. The following variables were assessed: right ventricular (RV) systolic pressure from tricuspid regurgitation (TR) jets, maximal velocity from pulmonary regurgitation jets, acceleration/ejection time ratio (AcT/ET) from the pulmonary flow, direction and maximal velocity across the VSD.

Bosentan was administered at a starting dose of 62.5 mg twice daily immediately after the baseline assessment. The dose was increased to the target dose of 125 mg twice daily 4 weeks later. Follow-up assessments were conducted in all patients at Months 3, 6, 9 and 12 of bosentan therapy and every 6 months, thereafter. Assessments included measurement of SpO_2_, blood tests, 6MWT and Doppler echo examination.

### Statistical analysis

Continuous variables are presented as means with standard deviations and ranges, categorical data are presented as counts and percentages. Statistical analyses were performed using the Wilcoxon signed-rank test. Variables were analysed at baseline and at sequential follow-up visits. A p value < 0.05 was considered as the level of significance.

## Results

In total, seven adults (four males, three females) with Down’s syndrome and ES were included. Their mean age was 29.6 ± 11.2 years, all patients were in WHO functional class III and had a VSD (two with associated mitral valve regurgitation). None of the patients had received previous PAH-specific therapy. Oxygen therapy was required for 10 hours/day by two patients. Concomitant therapy was required by six patients (thyroid hormones [n = 6], allopurinol [n = 3], ticlopidine [n = 1]). Baseline SaO_2_ at rest was 81.7 ± 6.6% (range 71–88%).

Patients were followed up for a mean period of 52.2 ± 3.9 months (range 46.0–55.5 months). All patients remained on bosentan monotherapy until the end of the study.

### Clinical assessment

In comparison with the baseline evaluation, at the final follow-up assessment, there was an improvement to WHO functional class II in one patient. Mean SaO_2_ at rest increased from 81.7 ± 6.6% (range 71–88%) at baseline to 89.0 ± 3% (range 87–92%) (*P* < 0.02) at Month 24 and 88.3 ± 3.2% (range 84–93%) at the final follow-up assessment (p < 0.05). AST and ALT levels increased slightly in one patient, but remained lower than twice the upper limit of normal.

**Figure 1:**
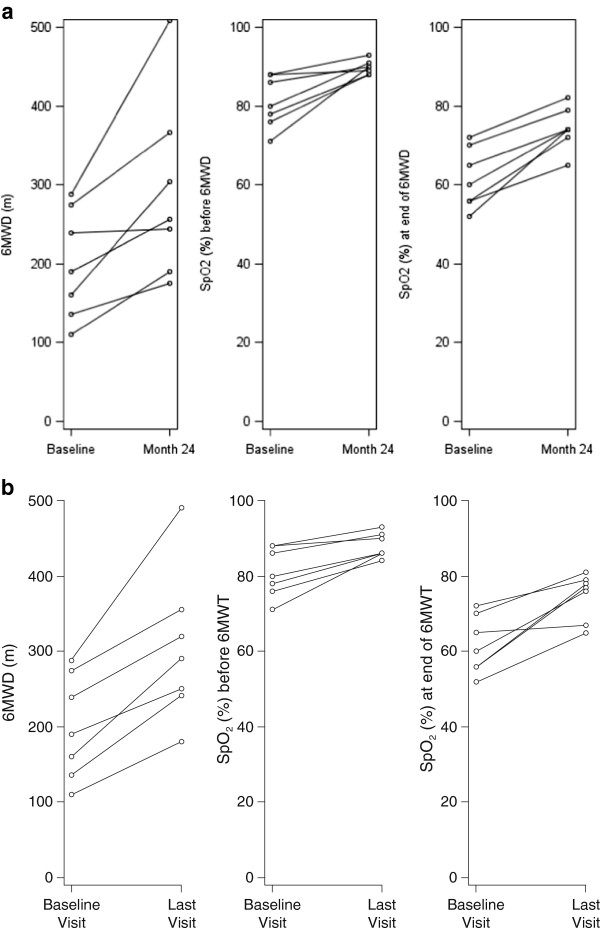
**6-minute walk distance (6MWD), peak exercise transcutaneous arterial oxygen saturation (SpO**_**2**_**) before and at the end of the 6-minute walk test at baseline and a. Month 24 and b. Last visit**

The mean 6MWD increased from 199.6 ± 69.1 metres at the baseline assessment to 291.9 ± 115 metres at Month 24 (*P* = 0.016) and 303.7 ± 99.9 metres at the final follow-up assessment (*P* = 0.016). The mean SpO_2_ at the end of the 6MWT was 61.6 ± 7.6% at the baseline assessment, 74.3 ± 5.4% at the Month 24 assessment (*P* = 0.016) and 74.7 ± 6.2% at the final follow-up assessments (p = 0.016). Borg dyspnoea index was 3.6 ± 1.4 at baseline and 2.4 ± 1.1 at the final follow-up assessment (not significant). Diastolic blood pressure decreased from 80.0 ± 5.8 mmHg (range: 70–90 mmHg) at the baseline assessment to 68.6 ± 4.8 mmHg (range: 60–75 mmHg) at the final assessment (Table 1, and Figure 1).

**Table 1:** Effect of bosentan on blood pressure, exercise capacity and blood oxygen saturation

**Parameter, mean ± SD (p value)**	**Baseline**	**Month 3**	**Month 6**	**Month 9**^**b**^	**Month 12**	**Month 18**	**Month 24**	**Final follow-up**^**a**^
SBP (mmHg)	125.7 ± 14.0	105.0 ± 11.8^b^ (0.063)	111.7 ± 9.8^b^ (0.125)	111.7 ± 5.2^b^ (0.094)	114.3 ± 13.7 (0.250)	113.7 ± 9.6 (0.188)	106.4 ± 7.5 (0.063)	108.6 ± 9.0 (0.094)
DBP (mmHg)	80.0 ± 5.8	74.2 ± 8.6^b^ (0.125)	79.2 ± 8.0^b^ (1.000)	73.3 ± 11.7^b^ (0.281)	69.3 ± 8.4 (0.031)	73.6 ± 8.5 (0.250)	68.9 ± 7.8 (0.063)	68.6 ± 4.8 (0.031)
6MWD (m)	199.6 ± 69.1	291.7 ± 73.1^b^ (0.063)	260.7 ± 60.2^b^ (0.063)	292.3 ± 112.7^b^ (0.031)	301.6 ± 88.7 (0.016)	317.4 ± 105.8 (0.016)	291.9 ± 115.4 (0.016)	303.7 ± 99.9 (0.016)
SaO_2_ (%) at rest	81.7 ± 6.6	87.2 ± 5.6^b^ (0.063)	88.4 ± 2.7 (0.031)	89.3 ± 2.5^b^ (0.031)	89.0 ± 2.5 (0.016)	87.9 ± 4.5 (0.109)	89.3 ± 1.8 (0.031)	88.3 ± 3.2 (0.016)
SpO_2_ (%) before 6MWT	81.0 ± 6.6	87.0 ± 6.4^b^ (0.094)	87.2 ± 3.8^b^ (0.031)	89.3 ± 1.0^b^ (0.031)	88.7 ± 3.1 (0.031)	88.6 ± 5.3 (0.047)	89.9 ± 1.8 (0.016)	88.0 ± 3.3 (0.016)
SpO_2_ (%) at end of 6MWT	61.6 ± 7.6	68.2 ± 9.3^b^ (0.250)	67.7 ± 3.0^b^ (0.063)	72.5 ± 6.2^b^ (0.031)	70.6 ± 10.1 (0.078)	71.0 ± 7.5 (0.016)	74.3 ± 5.4 (0.016)	74.7 ± 6.2 (0.016)
Borg dyspnoea index	3.6 ± 1.4	2.3 ± 1.2^b^ (0.063)	2.8 ± 1.5^c^ (0.250)	3.2 ± 1.2^b^ (1.000)	2.1 ± 1.2 (0.016)	2.9 ± 1.8 (0.313)	3.7 ± 2.9 (1.000)	2.4 ± 1.1 (0.250)

SBP: systolic blood pressure; DBP: diastolic blood pressure; 6MWT: 6-minute walk test; SaO_2_: transcutaneous arterial oxygen saturation; SpO_2_: peak exercise transcutaneous arterial oxygen saturation.

^a^Wilcoxon signed-rank test (two-sided) on change from baseline statistically significant (p < 0.05).

^b^n = 6 due to missing data.

^c^n = 5 due to missing data.

### Doppler echo assessment

Baseline echo parameters are shown in Table 2. At final follow-up there was a significant change in AcT (p < 0.0156) and AcT/ET ratio (p < 0.0156) from the pulmonary flow. RV and left ventricular systolic functions remained stable during follow-up (Table 2).

**Table 2:** Effect of treatment with bosentan on echocardiographic parameters

**Parameter**	**Baseline**	**Final follow-up**	***P *value**^**a**^
RVSP from TR (mmHg) (n = 4)	94.0 ± 13.4	85.5 ± 7.6	0.3750
Peak velocity of PR (m/s) (n = 2)	4.00 ± 0.14	4.05 ± 0.07	1.0000
AcT (m/s)	62.9 ± 11.6	83.0 ± 9.6	0.0156
AcT/ET	0.24 ± 0.04	0.30 ± 0.02	0.0156

RVSP: right ventricular systolic pressure; TR: tricuspid regurgitation; PR: pulmonary regurgitation; AcT: acceleration time; AcT/ET: acceleration/ejection time ratio; ^a^Wilcoxon signed-rank test.

## Discussion

In this long-term analysis, treatment of adult ES patients with Down’s syndrome with bosentan for a mean period of more than 4 years resulted in significant initial improvements in SaO_2_ at rest and 6MWD that were sustained during follow-up. Treatment options for patients with ES are limited, with the BREATHE-5 study being the only placebo-controlled study in this population [16]. It demonstrated that bosentan significantly reduced pulmonary vascular resistance index and mean pulmonary arterial pressure and significantly improved 6MWD in ES patients in WHO functional class III. Safety (change from baseline in SpO_2_) was the primary endpoint in the BREATHE-5 study and the authors demonstrated that bosentan did not worsen oxygen saturation. Data from a number of studies, including the current study, have since confirmed these benefits in the longer term in patients with PAH-CHD and specifically in patients with ES [19,21,25-28]. In addition, PAH-specific therapy has been shown to significantly lower the all-cause mortality rate in a large population of ES patients, providing strong conceptual support for treatment of all ES patients with PAH-specific therapy [29].

Patients with Down’s syndrome are at risk of developing PAH earlier than patients without Down’s syndrome and have worse functional capacity [1]. Both these factors are associated with poor long-term outcome [4,5,29-31]. There are no randomized controlled trials in this subpopulation of PAH-CHD patients. The results of our long-term analysis support data from previous open-label studies, which have demonstrated the efficacy of bosentan in ES patients with Down’s syndrome [19-21], and suggest that up to 4 years’ treatment can be beneficial in these patients. A previous study has shown that there was no significant difference in mortality between ES patients with and without Down’s syndrome after 4 years of bosentan treatment [21]. Similarly, no differences in the efficacy of bosentan between PAH-CHD patients with and without Down’s syndrome in a 12-month study have been reported, with both groups showing significant improvements in 6MWD, WHO functional class and haemodynamics [20].

The validity of the 6MWT in patients with Down’s syndrome has been questioned as it has been reported that level of intellectual disability is an independent factor for 6MWD [32]. Vis and coworkers showed that there was a significant difference in mean 6MWD between the group with a mild/moderate level of intellectual disability (318 ± 92 metres) and the group with a severe/profound level of intellectual disability (195 ± 84 metres). In our study, patients had a low level of intellectual disability; however, 6MWD was in the range seen in patients with severe intellectual disability in the Vis study. Importantly in the current study, patients achieved a significant increase in 6MWD with long-term bosentan therapy. These findings are similar to those from a recently published series of patients which demonstrated that 6MWD increased in patients with Down’s syndrome during the first 12 months of bosentan therapy – achieving early significance – and this improvement from baseline was maintained during a further 24 months of therapy [18]. Although it has been shown that there is no discrepancy between improvement in 6MWD and haemodynamic parameters in patients with Down’s syndrome [20], it is clear from the contrasting results from published small studies that we still need to understand more about use of the 6MWT in this population. Consequently, although there is some debate about the most appropriate assessment in this patient population, evidence of bosentan efficacy has been demonstrated. Based on our experience, we believe that patients’ compliance – in the presence of mild intellectual disability – is sufficient to guarantee a reliable result as confirmed by the assessment of objective parameters.

In the present study we did not perform an invasive evaluation such as right heart catheterization during follow-up visits. However, several studies report a high correlation (0.57 to 0.93) between echocardiographic and haemodynamic measurements of pulmonary arterial systolic pressure and, therefore, the echocardiographic assessment used in the current analysis provides valid information [33-35]. Baseline values of TR velocity and pulmonary arterial systolic pressure in the patients included in the current study were higher than those associated with the presence of pulmonary hypertension as specified in the current guidelines [24]. Additional variables to confirm the presence of pulmonary hypertension were also included in accordance with the guidelines (i.e. increased pulmonary valve regurgitation velocity, shortened acceleration time of RV ejection into the pulmonary artery, increased dimensions of the right heart chambers, abnormal shape and function of the interventricular septum and increased right ventricular wall thickness and pericardial effusion were demonstrated).

WHO functional class remained stable in the majority of patients during treatment, with an improvement in functional class being achieved by one patient. In the BREATHE-5 study, pulmonary vascular resistance index increased in the placebo arm during the 16-week study period, demonstrating the progressive nature of ES [16]. As a result, the maintenance of patients in WHO functional class III during prolonged bosentan therapy is encouraging.

The long-term results from this study provide confirmation of the significant improvement in exercise capacity and SpO_2_ in ES patients with Down’s syndrome seen in previous studies [17,20,21]. Additionally, this improvement was quick and progressive (in the first 6 months) and was then stable for up to 48 months.

Our study has some limitations, specifically the small number of patients and the lack of a placebo-controlled group and that data are only confirmed by echocardiography and not by right heart catheterization. However, it represents, with few other studies, one of the largest series of ES patients with Down’s syndrome treated with bosentan for an extended period. As a result the significant and stable increase in 6MWD and oxygen saturation are of clinical relevance.

## Conclusion

Long-term treatment with bosentan in adult ES patients with Down’s syndrome was well tolerated and significantly improved oxygen saturation and 6MWD. Our study confirms that the presence of Down’s syndrome does not affect the response to oral bosentan therapy in adult ES patients.

## Abbreviations

AcT/ET: Acceleration/ejection time ratio; ALT: Alanine aminotransferase; AST: Aspartate aminotransferase; AVSD: Atrioventricular septal defect; CHD: Congenital heart disease; echo: Echocardiography; ES: Eisenmenger’s syndrome; PAH: Pulmonary arterial hypertension; RV: Right ventricle; SaO2: Transcutaneous arterial oxygen saturation; SpO2: Peak exercise transcutaneous arterial oxygen saturation; TR: Tricuspid regurgitation; VSD: Ventricular septal defect; WHO: World Health Organization; 6MWD: 6-minute walk distance; 6MWT: 6-minute walk test.

## Competing interests

Roberto Crepaz, Cristina Romeo and Donato Montanaro disclose no potential conflicts of interest. Stefano De Santis is a full-time employee of Actelion Pharmaceuticals Italia Srl.

## Authors’ contributions

RC conceived the study, participated in its design and coordination. CR treated patients and performed evaluations. DM treated patients and performed evaluations. All authors contributed to the development of the manuscript and approved the final draft.

## Pre-publication history

The pre-publication history for this paper can be accessed here:

http://www.biomedcentral.com/1471-2261/13/74/prepub

## References

[B1] Galie N Manes A Palazzini M Negro L Marinelli A Gambetti S Mariucci E Donti A Branzi A Picchio FM Management of pulmonary arterial hypertension associated with congenital systemic-to-pulmonary shunts and Eisenmenger's syndrome Drugs 2008 68 1049 1066 10.2165/00003495-200868080-00004 18484798

[B2] Engelfriet PM Duffels MG Möller T Boersma E Tijssen JG Thaulow E Gatzoulis MA Mulder BJ Pulmonary arterial hypertension in adults born with a heart septal defect: the Euro Heart Survey on adult congenital heart disease Heart 2007 93 682 687 10.1136/hrt.2006.098848 17164490 PMC1955187

[B3] Duffels MG Engelfriet PM Berger RM van Loon RL Hoendermis E Vriend JW van der Velde ET Bresser P Mulder BJ Pulmonary arterial hypertension in congenital heart disease: an epidemiologic perspective from a Dutch registry Int J Cardiol 2007 120 198 204 10.1016/j.ijcard.2006.09.017 17182132

[B4] Van de Bruaene A Delcroix M Pasquet A De Backer J De Pauw M Naeije R Vachiéry JL Paelinck B Morissens M Budts W The Belgian Eisenmenger syndrome registry: implications for treatment strategies? Acta Cardiol 2009 64 447 453 10.2143/AC.64.4.2041608 19725436

[B5] Suzuki K Yamaki S Mimori S Murakami Y Mori K Takahashi Y Kikuchi T Pulmonary vascular disease in Down’s syndrome with complete atrioventricular septal defect Am J Cardiol 2000 86 434 437 10.1016/S0002-9149(00)00960-7 10946038

[B6] Morris JK Alberman E Trends in Down’s syndrome live births and antenatal diagnoses in England and Wales from 1989 to 2008: analysis of data from the National Down Syndrome Cytogenetic Register BMJ 2009 339 b3794 10.1136/bmj.b3794 19858532 PMC2767483

[B7] Mulder BJM Changing demographics of pulmonary arterial hypertension in congenital heart disease Eur Respir Rev 2010 19 308 313 10.1183/09059180.00007910 21119189 PMC9487491

[B8] Sherman SL Allen EG Bean LH Freeman SB Epidemiology of Down syndrome Ment Retard Dev Disabil Res Rev 2007 13 221 227 10.1002/mrdd.20157 17910090

[B9] Evans PR Cardiac anomalies in mongolism Br Heart J 1950 12 258 262 10.1136/hrt.12.3.258 15426687 PMC479394

[B10] Freeman SB Bean LH Allen EG Tinker SW Locke AE Druschel C Hobbs CA Romitti PA Royle MH Torfs CP Dooley KJ Sherman SL Ethnicity, sex, and the incidence of congenital heart defects: a report from the National Down Syndrome Project Genet Med 2008 10 173 180 10.1097/GIM.0b013e3181634867 18344706

[B11] Paladini D Tartaglione A Agangi A Teodoro A Forleo F Borghese A Martinelli P The association between congenital heart disease and Down syndrome in prenatal life Ultrasound Obstet Gynecol 2000 15 104 108 10.1046/j.1469-0705.2000.00027.x 10775990

[B12] Ferencz C Neill CA Boughman JA Congenital cardiovascular malformations with chromosome abnormalities: an epidemiologic study J Pediatr 1989 144 79 86 2521249 10.1016/s0022-3476(89)80605-5

[B13] Chéhab G El-Rassi I Abdo A Fakhoury H Chokor I Haddad W Saliba Z Atrioventricular septal defect characteristics in infants with and without Down's syndrome: a Lebanese study J Med Leban 2010 58 3 7 20358852

[B14] Placidi S Digilio MC Marino B Types of cardiac defects in children with Down’s syndrome Cardiol Young 2006 16 198 199 10.1017/S1047951106220225 16553989

[B15] Elmagrpy Z Rayani A Shah A Habas E Aburawi EH Down syndrome and congenital heart disease: why the regional difference as observed in the Libyan experience? Cardiovasc J Afr 2011 22 306 309 10.5830/CVJA-2010-072 22159317 PMC3721875

[B16] Galiè N Beghetti M Gatzoulis MA Granton J Berger RM Lauer A Chiossi E Landzberg M Bosentan randomized trial of endothelin antagonist therapy-5 (BREATHE-5) investigators Bosentan therapy in patients with Eisenmenger Syndrome: a multicenter, double-blind, randomized, placebo-controlled study Circulation 2006 114 48 54 10.1161/CIRCULATIONAHA.106.630715 16801459

[B17] Duffels MGJ Vis JC van Loon RLE Berger RM Hoendermis ES van Dijk AP Bouma BJ Mulder BJ Down patients with Eisenmenger syndrome: is bosentan treatment an option? Int J Cardiol 2009 134 378 383 10.1016/j.ijcard.2008.02.025 18579234

[B18] Serino G Guazzi M Micheletti A Lombardi C Danesi R Negura D Carminati M Chessa M Effect of bosentan on exercise capacity and clinical worsening in patients with dual down and eisenmenger syndrome Clin Med Insights Cardiol 2013 7 29 34 23440179 10.4137/CMC.S10237 PMC3572875

[B19] Monfredi O Griffiths L Clarke B Mahadevan VS Efficacy and safety of bosentan for pulmonary arterial hypertension in adults with congenital heart disease Am J Cardiol 2011 108 1483 1488 10.1016/j.amjcard.2011.07.006 21943933

[B20] D'Alto M Romeo E Argiento P D'Andrea A Sarubbi B Correra A Scognamiglio G Papa S Bossone E Calabrò R Vizza CD Russo MG Therapy for pulmonary arterial hypertension due to congenital heart disease and Down's syndrome Int J Cardiol 2013 164 64 69 10.1016/j.ijcard.2011.06.064 21802156

[B21] Vis JC Duffels MG Mulder P de Bruin-Bon RH Bouma BJ Berger RM Hoendermis ES van Dijk AP Mulder BJ Prolonged beneficial effect of bosentan treatment and 4-year survival rates in adult patients with pulmonary arterial hypertension associated with congenital heart disease Int J Cardiol 2013 164 64 69 10.1016/j.ijcard.2011.06.064 21723630

[B22] Kermeen FD Franks C O'Brien K Seale H Hall K McNeil K Radford D Endothelin receptor antagonists are an effective long term treatment option in pulmonary arterial hypertension associated with congenital heart disease with or without trisomy 21 Heart Lung Circ 2010 19 595 600 10.1016/j.hlc.2010.07.005 20728407

[B23] Wechsler D The Measurement and Appraisal of Adult Intelligence 1958 4 Baltimore, MD, USA: Williams & Wilkins Co 297 doi: 10.1037/11167-000

[B24] Galiè N Hoeper MM Humbert M Torbicki A Vachiery JL Barbera JA Beghetti M Corris P Gaine S Gibbs JS Gomez-Sanchez MA Jondeau G Klepetko W Opitz C Peacock A Rubin L Zellweger M Simonneau G Vahanian A Auricchio A Bax J Ceconi C Dean V Filippatos G Funck-Brentano C Hobbs R Kearney P McDonagh T McGregor K Popescu BA Guidelines for the diagnosis and treatment of pulmonary hypertension: the task force for the diagnosis and treatment of pulmonary hypertension of the European Society of Cardiology (ESC) and the European Respiratory Society (ERS), endorsed by the International Society of Heart and Lung Transplantation (ISHLT) Eur Heart J 2009 30 2493 2537 19713419 10.1093/eurheartj/ehp297

[B25] D'Alto M Vizza CD Romeo E Badagliacca R Santoro G Poscia R Sarubbi B Mancone M Argiento P Ferrante F Russo MG Fedele F Calabrò R Long term effects of bosentan treatment in adult patients with pulmonary arterial hypertension related to congenital heart disease (Eisenmenger physiology): safety, tolerability, clinical, and haemodynamic effect Heart 2007 93 621 625 10.1136/hrt.2006.097360 17135220 PMC1955562

[B26] Diller GP Dimopoulos K Kaya MG Harries C Uebing A Li W Koltsida E Gibbs JS Gatzoulis MA Long-term safety, tolerability and efficacy of bosentan in adults with pulmonary arterial hypertension associated with congenital heart disease Heart 2007 93 974 976 10.1136/hrt.2006.089185 17639112 PMC1994431

[B27] Díaz-Caraballo E González-García AE Reñones M Sánchez-Recalde A García-Río F Oliver-Ruiz JM Long-term bosentan treatment of complex congenital heart disease and Eisenmenger's syndrome Rev Esp Cardiol 2009 62 1046 1049 10.1016/S0300-8932(09)72103-7 19712626

[B28] Kaya MG Lam YY Erer B Ayhan S Vatankulu MA Nurkalem Z Meric M Eren M Eryol NK Long-term effect of bosentan therapy on cardiac function and symptomatic benefits in adult patients with Eisenmenger syndrome J Card Fail 2012 18 379 384 10.1016/j.cardfail.2012.02.004 22555267

[B29] Dimopoulos K Inuzuka R Goletto S Giannakoulas G Swan L Wort SJ Gatzoulis MA Improved survival among patients with Eisenmenger syndrome receiving advanced therapy for pulmonary arterial hypertension Circulation 2010 121 20 25 10.1161/CIRCULATIONAHA.109.883876 20026774

[B30] Chi TP Krovetz JL The pulmonary vascular bed in children with Down syndrome J Pediatr 1975 86 533 538 10.1016/S0022-3476(75)80142-9 123955

[B31] Yamaki S Yasui H Kado H Yonenaga K Nakamura Y Kikuchi T Ajiki H Tsunemoto M Mohri H Pulmonary vascular disease and operative indications in complete atrioventricular canal defect in early infancy J Thorac Cardiovasc Surg 1993 106 398 405 8361179

[B32] Vis JC Thoonsen H Duffels MG de Bruin-Bon RA Huisman SA van Dijk AP Hoendermis ES Berger RM Bouma BJ Mulder BJ Six-minute walk test in patients with Down syndrome: validity and reproducibility Arch Phys Med Rehabil 2009 90 1423 1427 10.1016/j.apmr.2009.02.015 19651279

[B33] Bossone E Avelar E Bach DS Gillespie B Rubenfire M Armstrong WF Diagnostic value of resting tricuspid regurgitation velocity and right ventricular ejection flow parameters for the detection of exercise-induced pulmonary arterial hypertension Int J Card Imaging 2000 16 429 436 10.1023/A:1010604913656 11482708

[B34] Denton C Cailes J Phillips G Wells A Black C Du Bois R Comparison of Doppler echocardiography and right heart catheterization to assess pulmonary hypertension in systemic sclerosis Br J Rheumatol 1997 36 239 243 10.1093/rheumatology/36.2.239 9133938

[B35] Penning S Robinson K Major C Garite T A comparison of echocardiography and pulmonary artery catheterization for evaluation of pulmonary artery pressures in pregnant patients with suspected pulmonary hypertension Am J Obstet Gynecol 2001 184 1568 1570 10.1067/mob.2001.114857 11408882

